# Common variants upstream of *KDR* encoding VEGFR2 and in *TTC39B* associate with endometriosis

**DOI:** 10.1038/ncomms12350

**Published:** 2016-07-25

**Authors:** Valgerdur Steinthorsdottir, Gudmar Thorleifsson, Kristrun Aradottir, Bjarke Feenstra, Asgeir Sigurdsson, Lilja Stefansdottir, Anna M. Kristinsdottir, Florian Zink, Gisli H. Halldorsson, Nete Munk Nielsen, Frank Geller, Mads Melbye, Daniel F. Gudbjartsson, Reynir T. Geirsson, Unnur Thorsteinsdottir, Kari Stefansson

**Affiliations:** 1 deCODE Genetics/Amgen, 101 Reykjavik, Iceland; 2 Department of Obstetrics and Gynecology, Landspitali University Hospital, 101 Reykjavik, Iceland; 3 Department of Epidemiology Research, Statens Serum Institut, DK-2300 Copenhagen, Denmark; 4 Department of Clinical Medicine, University of Copenhagen, DK-2100 Copenhagen, Denmark; 5 Department of Medicine, Stanford University School of Medicine, Stanford, CA 94305, USA; 6 School of Engineering and Natural Sciences, University of Iceland, 101 Reykjavik, Iceland; 7 Faculty of Medicine, School of Health Sciences, University of Iceland, 101 Reykjavik, Iceland

## Abstract

We conducted a genome-wide association scan (GWAS) of endometriosis using 25.5 million sequence variants detected through whole-genome sequencing (WGS) of 8,453 Icelanders and imputed into 1,840 cases and 129,016 control women, followed by testing of associated variants in Danish samples. Here we report the discovery of a new endometriosis susceptibility locus on 4q12 (rs17773813[G], OR=1.28; *P*=3.8 × 10^−11^), upstream of *KDR* encoding vascular endothelial growth factor receptor 2 (VEGFR2). The variant correlates with disease severity (*P*=0.0046) when moderate/severe endometriosis cases are tested against minimal/mild cases. We further report association of rs519664[T] in *TTC39B on 9p22* with endometriosis (*P*=4.8 × 10^−10^; OR=1.29). The involvement of *KDR* in endometriosis risk highlights the importance of the VEGF pathway in the pathogenesis of the disease.

## Abstract (web summary)

Endometriosis is a common gynaecological disease but the underlying pathogenesis is poorly understood, however there is a strong familial component. Here the authors conduct a genome wide association study and identify a novel susceptibility locus that correlates with disease severity.

## Graphical abstract

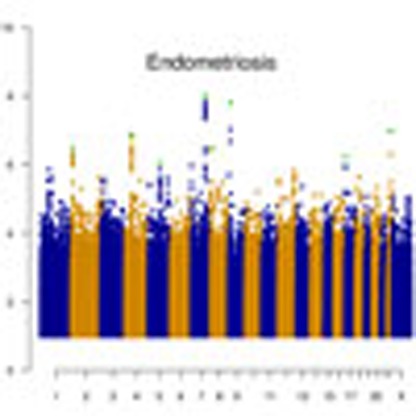

Endometriosis is an oestrogen-dependent condition characterized by the presence of endometrial tissue outside the uterine cavity. It is one of the most common gynaecologic diseases, with an estimated prevalence of 10% during reproductive age. In women with fertility problems the prevalence is even higher but with variable estimates between studies ranging from 35 to 50% (refs 1, 2). Endometriosis may be asymptomatic or accompanied by pain and/or infertility. The American Fertility Society (rAFS) classifies severity of endometriosis into four stages (I-minimal, II-mild, III-moderate and IV-severe) depending on location, extent and depth of endometriosis implants; presence and severity of adhesions; and presence and size of ovarian endometriomas^3^. The understanding of the pathogenesis of endometriosis is relatively poor but the current thinking is that the underlying cause is retrograde menstruation leading to implantation of exfoliated endometrium in the pelvic cavity. However, retrograde menstruation is frequently observed in healthy women^4^,^5^. Hence, other factors are clearly important.

Family based and twin studies have demonstrated a strong familial component to endometriosis^6^,^7^,^8^. The genetic burden has been shown to correlate with rAFS stages indicating that this classification could be useful for genetic studies^9^. Since the arrival of genome-wide association scan (GWAS) several groups have reported variants that associate with endometriosis in individuals of European and Japanese origin^10^,^11^,^12^,^13^. A recent meta-analysis of 11 single nucleotide polymorphisms (SNPs) at nine loci, reported in the GWAS studies, found that seven SNPs at six loci reached genome-wide significance^14^. While previous studies were limited to variants on genome-wide SNP arrays, here we report results of association with endometriosis of more than 25 million sequence variants identified through whole-genome sequencing (WGS) of 8,453 Icelanders and imputed into a large fraction of the Icelandic population. This data set provides both more complete coverage of sequence variants in the genome and allows rare variants, reliably imputed, to be tested for association.

In this study we found two new loci that associate with endometriosis. One of the loci is upstream of *KDR*, implicating the VEGF pathway in the pathogenesis of endometriosis.

## Results

### GWAS analysis

We included in the GWAS 1,840 women surgically diagnosed with endometriosis, of which 688 were diagnosed as stage III/IV, and 129,016 women as controls. The 25.5 million sequence variants (20.6 million single nucleotide variants and 4.9 million short insertions-deletions (INDELs); all with imputation info>0.8) tested for association with endometriosis using logistic regression under the multiplicative model, were detected through high coverage WGS (median depth of 32 ×) of 8,453 Icelanders and subsequently imputed into those who had been genotyped with Illumina SNP arrays and their first and second-degree relatives (Methods section). The threshold for genome-wide significance was corrected for multiple testing using a weighted Bonferroni procedure based on functional impact of classes of variants^15^ (Methods section).

In the scan, 9 out of 11 previously reported variants replicated nominally (*P*<0.05) and for all variants the directions of effects were consistent with those reported (Supplementary Table 1). Of these variants the strongest association was with the *GREB1* splice acceptor variant rs13394619 (*P*=7.4 × 10^−7^; odds ratio (OR)=1.20).

### Novel loci associated with endometriosis

No variant reached the threshold of genome-wide significance for association with endometriosis (Supplementary Fig. 1), but eight loci associated with *P* value<1 × 10^−6^, including the previously reported *GREB1* locus and seven novel loci (Supplementary Fig. 1; Supplementary Table 2). One of these markers, a rare single-base-pair deletion (delC), rs767233639 (minor allele frequency (MAF)=0.05%) in the 3′UTR of *RTN4RL1* (*P*=6.0 × 10^−7^; OR=9.19), was neither present in the Exome Aggregation Consortium (http://exac.broadinstitute.org) nor in the Exome Variant Server (http://evs.gs.washington.edu/EVS/; v0017 2012; November) indicating that it is not present in European populations outside Iceland.

For validation, we tested peak markers at the remaining six loci for association with endometriosis in 514 Danish cases and 749 controls. Two of the markers replicated in the Danish samples, rs17773813 located 17 kb upstream of *KDR* and an intronic variant, rs519664, in *TTC39B* (Supplementary Table 2). In Iceland, rs17773813[G] associates with endometriosis with an OR=1.24 and a *P*=1.3 × 10^−7^ and in Denmark with an OR=1.49 and a *P*=1.3 × 10^−5^ (Table 1). After combining the Icelandic and the Danish data the association reached genome-wide significance with *P*=3.8 × 10^−11^ and OR=1.28 (Table 1). In a previous report^12^ suggestive association of rs4241991, located immediately downstream of KDR, with endometriosis was reported. This variant is not in LD with rs17773813 and does not associate with endometriosis in our data (*P*=0.72). The *TTC39B* intronic variant rs519664[T] associates with endometriosis in Iceland with an OR=1.28 and a *P*=1.6 × 10^−8^ and in Denmark with an OR=1.30 and a *P*=0.0090 (Table 1). Combined the association is genome-wide significant with *P*=4.8 × 10^−10^ and OR=1.29 (Table 1).

When testing the association of rs17773813[G] in Iceland using only the stage III/IV endometriosis cases, the OR was 1.47 and *P*=1.4 × 10^−8^ but no association was found with stage I/II endometriosis (*P*=0.33; OR=1.07), suggesting that this variant associates with the development of moderate to severe disease (Table 1). We tested this further by directly contrasting the 688 stage III/IV cases and 620 cases with stage I/II disease. The results, *P*=0.0046 further support the notion that there is a correlation of this variant with disease severity (Table 1). For rs519664[T] the association with stage III/IV disease was *P*=1.9 × 10^−5^ and OR=1.35 and with stage I/II *P*=0.013 and OR=1.21 (Table 1). The difference between stage III/IV and stage I/II disease was not significant (*P*=0.18). Unfortunately, information on disease severity is not available for the Danish data set so further work is needed to confirm the effect of these variants on disease severity.

*KDR* is the strongest candidate gene for the rs17773813[G] signal based on the proximity to the variant and the biology of the protein products. It encodes the vascular endothelial growth factor receptor 2 (VEGFR2), the main VEGF receptor on endothelial cells. The VEGF/VEGFR signalling pathways have been characterized as central processes for angiogenesis^16^,^17^,^18^ where VEGFR2 is the major signal transducer^19^. The role of VEGF in endometriosis has been extensively studied and expression of VEGF in eutopic and ectopic endometrium compared. However, fewer studies have directly addressed the role of the VEGF receptors in endometriosis. Higher expression of VEGFR2 in blood vessels from eutopic endometrium of women with endometriosis than from with women without endometriosis has been reported^20^. There are two functionally distinct protein products encoded by KDR (refs 21, 22). The full-length VEGFR2, consists of 7 immunoglobulin domains, a transmembrane domain and tyrosine kinase domains and is the main angiogenic receptor for vascular endothelial growth factor A (VEGFA) (ref. 19). The smaller isoform, soluble VEGFR2 (sVEGFR2), is lacking the tyrosine kinase domain as well as the transmembrane domain and acts as a negative regulator of VEGF function^22^.

**Figure 1:**
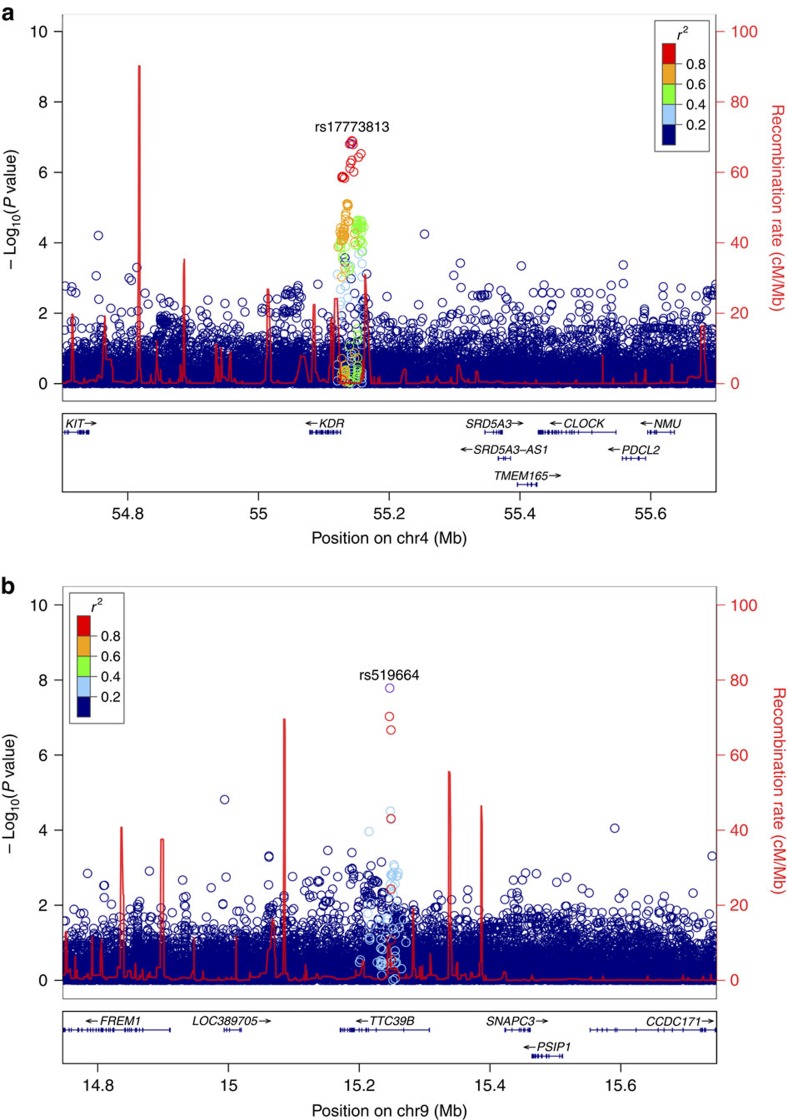
Locus plot for *KDR* and *TTC39B* loci in endometriosis. Data are based on association with all endometriosis (expressed as −log_10_(*P* value) derived by logistic regression) for variants identified by WGS and imputation. Variants are plotted in colours according to their *r*^2^ values relative to the index SNP as indicated. Recombination rates, in cM/Mb, estimated from the International HapMap Project are plotted as red lines. The lower panel sections shows the locations of RefSeq genes and the chromosomal position (NCBI hg38, Build 38). The region shown is ±500 kb from the index SNP. (**a**) *KDR* locus on chr4q12; index SNP rs17773813. (**b**) *TTC39B* locus on chr9p22; index SNP rs519664.

Rs17773813 is one of 19 highly correlated variants (all with *R*^2^>0.9 with rs17773813) located in a 20 kb region upstream of *KDR* (Fig. 1a). Most of these variants are predicted to affect sequence motifs and some have additional hallmarks of regulatory elements (HaploReg v4.1)^23^ and therefore could have a role in the regulation of neighbouring genes. However, we find no correlation of any of these variants with expression of *KDR* or other genes in the region in the uterus or 43 other different tissues reported in the genotype-tissue expression (GTEx) Portal (GTEx V6)^24^. In these data, the transcript that encodes the soluble form is not detected, only that for membrane bound receptor.

The intronic variant rs519664 is located in intron 2 of *TTC39B,* a gene that spans 135 kb encoding a potential transmembrane protein of unknown function (Fig. 1b). Other uncorrelated variants in this gene have been associated with lipids in GWAS studies^25^. Like rs17773813 near *KDR*, rs519664 does not correlate with expression of *TTC39B* or other genes in the region in the reported tissues (GTEx V6). Interestingly, a region close to rs519664 has recently been shown to be a putative regulatory element that physically interacts with the promoters of nearby genes as well as an alternative promoter of *TTC39B* (ref. 26).

### Suggestive novel locus on chromosome 17

Since the rare marker in *RTN4RL1* on chromosome 17 is absent from Danes, we searched for additional evidence for its association with endometriosis in the Icelandic samples. We found that in Iceland it appears to associate with severe endometriosis (stage III/IV) with *P*=7.9 × 10^−7^ and OR=15.45 but not with the milder stage I/II (*P*=0.067; OR=5.19; Table 1). However, when we directly tested stage III/IV cases against stage I/II the difference was not significant (*P*=0.27).

*RTN4RL1* encodes Nogo receptor 3 (NgR3), a receptor for chondroitin sulfate proteoglycans^27^. Proteoglycans play a major role in the tissue architecture and extracellular matrix organization of the endometrium where they also play a role in inhibition of cell proliferation^28^. However, there is no direct biological evidence to support a role for NgR3 in the endometrium.

Although the strongest association on chromosome 17 is with rs767233639 in *RTN4RL1* the association signal spans a large region (∼5 Mb) (Supplementary Fig. 2; Supplementary Table 3), which is commonly seen for rare signals. Conditional analysis demonstrated that after adjusting for rs767233639 the association with other variants vanished, however, when adjusting for the other variants, rs767233639 remained nominally associated (Supplementary Table 4). The region spanning the associations was well covered in our sequence data (Supplementary Fig. 2). However, one cannot exclude the possibility that the causative variant is not part of the data set used in this study and genes in the region other than *RTN4RL1* may be the culprit at this locus.

## Discussion

In spite of extensive evidence for the importance of angiogenesis in the development of endometriosis, this is the first time a sequence variant by a gene that plays a key role in angiogenesis is shown to associate with endometriosis. Angiogenic factors such as VEGF, other growth factors and their receptors that are involved in the progression of endothelial cells are thought to play a role in the development of endometriosis. Bourlev *et al.*^20^ found significantly higher micro-vessel density in the secretory phase of eutopic endometrium from women with endometriosis, than from women without endometriosis. Furthermore, this was accompanied by a significantly greater expression of VEGFA in glandular epithelium and of VEGFR2 in blood vessels as well as a higher level of VEGFA in peritoneal fluid. They concluded that there is a more pro-angiogenic environment in eutopic endometrium from women with endometriosis than from women without the disease. Furthermore, vascular density and the expression of both VEGF and VEGFR2 are significantly higher in deep infiltrating endometriotic lesions than in eutopic endometrium^29^.

The association signal at the *KDR* locus is comprised of a cluster of highly correlated variants in a 20 kb region upstream of *KDR*. Although we were unable to find evidence for an effect of those variants on *KDR* expression in 44 tissues in the GTEx database, the overlap of most of those variants with regulatory elements suggests that one or more of them may affect some regulatory function. For endometriosis the most relevant tissue is the endometrium that is currently not present in GTEx. Another limitation of this data set is that it only includes the transcript encoding the membrane bound VEGFR2. However, any dysregulation of the ratio between the membrane bound and the soluble form of the protein could be important as they have opposite effects on VEGF signalling. Increased expression of the membrane bound VEGFR2 or reduced expression of the soluble form could thus both lead to higher angiogenic capacity in the ectopic endometrium.

The ORs we report for the *KDR* variant 1.28 and the *TTC39B* variant 1.29 (Table 1) are at least nominally higher than the meta-analysis based ORs reported for other endometriosis associated variants (Supplementary Table 1)^14^. Most of the reported variants show higher OR in stage III/IV cases than if all endometriosis cases are included (Supplementary Table 1)^14^. This is also true for the *KDR* and *TTC39B* variants. Moreover, we also find no evidence for association of the *KDR* variant with stage I/II endometriosis in our data. Perhaps this reflects a role of angiogenesis in the development of deeper and more severe endometriosis implants. Furthermore, the association of a variant near *KDR* reinforces the suspected role of angiogenesis in the pathogenesis of this enigmatic trait.

## Methods

### Study populations

The Icelandic cases were women with pelvic endometriosis diagnosed at laparoscopy or laparotomy in the years 1981–2010 at gynecology departments in Iceland^8^,^30^. Information on diagnosis was obtained: (i) by searching the national computerized database of hospital admissions for the relevant diagnostic codes, International Classification of Diseases (ICD)-8, code 625.3; ICD-9, codes 617.0–617.9; and ICD-10, codes N80.0–80.9; (ii) from local paper databases with diagnostic codes and operation-theater registers from smaller hospitals and two private clinics not linked to the centralized registry; (iii) from the centralized, computerized, country-wide Pathology Registry at Landspitali, Systematized Nomenclature of Medicine system (SNOMED) code 76500. Individual operation notes were examined for each patient to confirm the diagnosis. The revised American Society for Reproductive Medicine (rAFS) classification^3^ was used, and cases were classified as stage I–II (minimal/mild) or stage III–IV (moderate/severe). Staging was based on both operative notes and pathology records where relevant. rAFS staging is available for over 70% of the cases. Controls were women recruited through different genetic research projects at deCODE. Women with known endometriosis diagnosis were excluded from the control group. All participants gave informed consent and the study was approved by the Data Protection Commission of Iceland and the National Bioethics Committee of Iceland.

The replication samples were drawn from the Danish National Birth Cohort (DNBC), a population-based cohort of more than 100,000 pregnancies, recruited in the years 1996–2002 (ref. 31). Extensive phenotype information is available for the DNBC mothers and children based on computer-assisted telephone interviews, questionnaire-based follow-up surveys and data from Danish population and health registers. DNBC women with endometriosis were identified from the Danish National Patient Register^32^, which includes information on all inpatient admissions since 1977 and all emergency and outpatient hospital contacts since 1995, with diagnostic information coded according to the ICD (version 8 through 1993 and version 10 from 1994 onwards). Endometriosis was defined using ICD8 codes 625.30-625.39 and ICD10 code group N80. To ensure a high degree of genetic homogeneity in the genotyped sample, we obtained birthplace information from the Civil Registration System^33^ and only included women who themselves as well as their parents were born in Scandinavia. After genotyping, a total of 514 cases and 749 controls from the DNBC with at least 90% genotyping yield were included in the analysis. The study was approved by the Scientific Ethics Committee for the Capital City Region (Copenhagen) and the Danish Data Protection Agency. The Scientific Ethics Committee also granted exemption from obtaining informed consent from participants (H-15000244) as the study was based on biobank material.

### GWAS genotyping and imputation

Genotyping and imputation methods in the Icelandic samples were based on WGS, chip genotyping and long-range phasing of Icelandic population samples^34^. We sequenced the whole genomes of 8,453 Icelanders using Illumina technology to a mean depth of at least 10 × (median 32 ×). The sequencing was performed using the following three different library preparation methods and sequencing instruments from Illumina: (i) standard TruSeq DNA library preparation method; Illumina GAIIx and/or HiSeq 2000 sequencers; (ii) TruSeq DNA PCR-free library preparation method; Illumina HiSeq 2500 sequencers; and (iii) TruSeq Nano DNA library preparation method; Illumina HiSeq X sequencers. SNPs and indels were identified and their genotypes called using joint calling with the Genome Analysis Toolkit HaplotypeCaller (GATK version 3.3.0)^35^. Genotype calls were improved by using information about haplotype sharing, taking advantage of the fact that all the sequenced individuals had also been chip-typed and long-range phased. The sequence variants identified in the 8,453 sequenced Icelanders were then imputed into 150,656 Icelanders who had been genotyped with various Illumina SNP chips and their genotypes phased using long-range phasing^36^,^37^. Using genealogic information, the sequence variants were imputed into 294,212 un-typed relatives of the chip-typed to further increase the sample size for association analysis and increased the power to detect associations.

### Association analysis

Women with endometriosis and female controls were either chip-typed (1,379 cases; 68,890 controls) or first-/second-degree relatives of chip-typed individuals that were not chip-typed themselves (461 cases; 60,126 controls). Association testing for case–control analysis was performed using logistic regression, adjusting for age and county. A total of 25.5 million variants with MAF>0.03%, (20.6 million SNPs and 4.9 million short INDELs) were used in the association analysis under a multiplicative model. All of the variants that were tested had imputation information over 0.8. Test for association in the Danish replication samples was done using logistic regression implemented in the NEMO software^38^. Results from the discovery and replication cohorts were combined using a Mantel–Haenszel model^39^. All *P* values presented are based on logistic regression.

The threshold for genome-wide significance association in the Icelandic samples was corrected for multiple testing using a class-specific Bonferroni procedure based on functional impact of classes of variants^15^. This yielded significance thresholds of 3.3 × 10^−7^ for 5,969 high-impact variants (including stop-gained, frameshift, splice acceptor or donor), 6.5 × 10^−8^ for 118,721 moderate-impact variants (including missense, splice-region variants and in-frame INDELs), 5.9 × 10^−9^ for 1,797,313 low-impact variants (including synonymous variants 3′ and 5′ UTR variants), 3.0 × 10^−9^ for 3,302,617 intergenic and deep intronic variants overlapping DNase hypersensitivity sites and 9.9 × 10^−10^ for 20,252,868 other variants (intergenic and deep intronic).

To account for inflation in test statistics due to cryptic relatedness and stratification within the case and control sample sets, we applied the method of LD score regression^40^. With a set of 1.1million variants we regressed the *χ*^2^ statistics from our GWAS scan against LD score and used the intercept as a correction factor. The LD scores were downloaded from a LD score database (ftp://atguftp.mgh.harvard.edu/brendan/1k_eur_r2_hm3snps_se_weights.RDS) accessed on 23 June 2015. The estimated correction factor was 1.07 and 1.09 for endometriosis all and endometriosis stage III/IV, respectively. The relationship between LD score regression and genomic control inflation factor for the endometriosis data set is shown in Supplementary Fig 3.

### Single-variant genotyping

Single-SNP genotyping was carried out applying the Centaurus (Nanogen) platform^41^. The rare indel in *RTN4RL1* rs767233639 was genotyped by Sanger sequencing. Imputation of rs767233639 was validated by direct genotyping of 58 carriers and 77 non-carriers. Good correlation was found between genotyped and imputed genotypes (*R*^2^>0.99).

### Data availability

The Icelandic population WGS data has been deposited at the European Variant Archive under accession code PRJEB8636.

The authors declare that the data supporting the findings of this study are available within the article, its supplementary Information files and on request.

## Additional information

**How to cite this article**: Steinthorsdottir, V. *et al.* Common variants upstream of KDR encoding VEGFR2 and in TTC39B associate with endometriosis. *Nat. Commun.* 7:12350 doi: 10.1038/ncomms12350 (2016).

## Supplementary Material

Supplementary Information Supplementary Figures 1-3 and Supplementary Tables 1-4

## Figures and Tables

**Table 1:** Association of sequence variants near *KDR* and in *TTC39B* and *RTN4RL1* with endometriosis.

**SNP**	**Risk allele**	**Chr**	Position*	**Nearest gene**	**Sample set**	**Number of cases**	**Number of controls**	**RAF (%)**†	**OR**	**95% CI**	***P*** **value**
rs17773813	G	4	55142802	*KDR*	Iceland endometriosis all	1,840	129,016	68.2	1.24	(1.14–1.34)	1.3 × 10^−7^
					Denmark endometriosis all	514	749	68.0	1.49	(1.25–1.78)	1.3 × 10^−5^
					Combined	2,354	129,765	–	1.28	(1.19–1.38)	3.8 × 10^−11^
					Iceland stage I/II	620	127,943	68.2	1.07	(0.93–1.23)	0.33
					Iceland stage III/IV	688	123,526	68.2	1.47	(1.29–1.68)	1.4 × 10^−8^
					Stage III/IV versus I/II	688	620	–	1.32	(1.09–1.60)	4.6 × 10^−3^
											
rs519664	T	9	15246654	*TTC39B*	Iceland endometriosis all	1,840	129,016	20.4	1.28	(1.17–1.39)	1.6 × 10^−8^
					Denmark endometriosis all	514	749	18.2	1.30	(1.07–1.58)	0.0090
					Combined	2,354	129,765	–	1.29	(1.19–1.39)	4.8 × 10^−10^
					Iceland stage I/II	620	127,943	20.4	1.21	(1.04–1.40)	0.013
					Iceland stage III/IV	688	123,526	20.4	1.35	(1.18–1.55)	1.9 × 10^−5^
					Stage III/IV versus I/II	688	620	–	1.15	(0.94–1.41)	0.18
											
rs767233639	delC	17	1934814	*RTN4RL1*	Iceland endometriosis all	1,840	129,016	0.05	9.19	(3.85–21.96)	6.0 × 10^−7^
					Iceland stage I/II	620	127,943	0.05	5.19	(0.91–31.07)	0.067
					Iceland stage III/IV	688	123,526	0.05	15.45	(5.21–45.81)	7.9 × 10^−7^
					Stage III/IV versus I/II	688	620	–	2.12	(0.56–8.06)	0.27

Chr, chromosome; RAF, risk allele frequency; CI, confidence interval.

^*^NCBI hg38 Build 38.

^†^Risk allele frequency in controls
