# Supplementary material for: Common variants upstream of KDR encoding VEGFR2 and in TTC39B associate with endometriosis
Source: Nat Commun. 2016 Jul 25;7:12350. doi: 10.1038/ncomms12350 (PMC4962463; doi:10.1038/ncomms12350)
Supplement: Supplementary Information — Supplementary Figures 1-3 and Supplementary Tables 1-4 [file ncomms12350-s1.pdf]

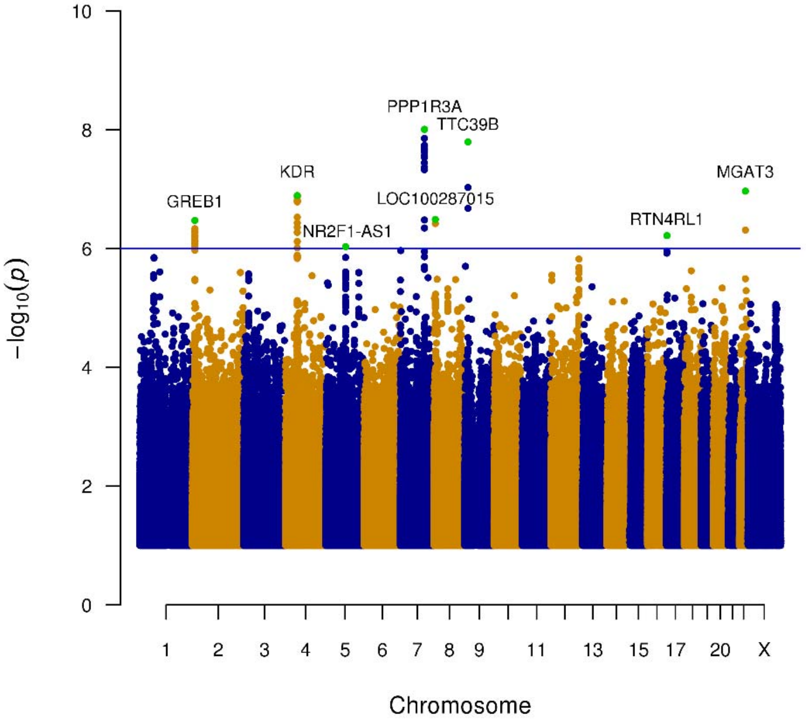

Supplementary Figure 1. Manhattan plot showing genome wide association results for all endometriosis

Results are shown for variants with association *P*-value < 0.1 and imputation information > 0.9. Variants selected for follow up are highlighted in green and labels indicate the nearest gene (see Supplementary Table 2).

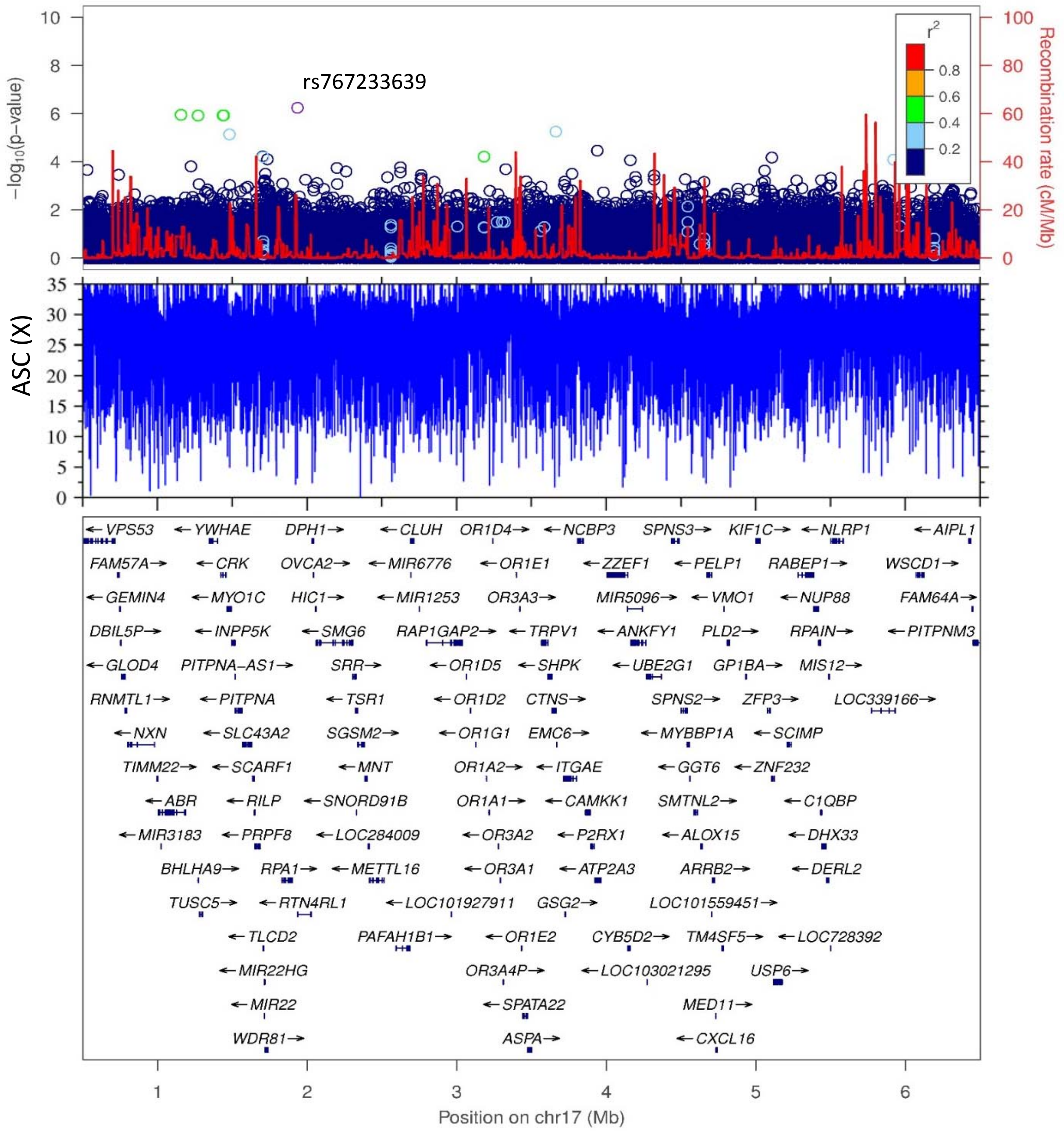

Supplementary Figure 2 . Regional plot of chr17p showing rs767233639 and correlated variants with evidence of associaton with endometriosis

Regional association plot for a 6 Mb region on chromosome 17 showing variants correlated with rs767233639 ($R^2 > 0.2$) and with evidence of associaton with endometriosis ($P < 0.01$). The top panel shows the -$\log_{10}$ *P*-values (left vertical axis) for variants association with endometriosis in the Icelandic samples against their position, together with estimates of recombination rate from the International HapMap Project (right vertical axis). The second panel shows the average sequence coverage in the 8,453 WGS individuals across the region. The lowest panel shows known genes in the region, taken from the UCSC genome browser. Note that due to the size of the region not all genes are shown. All positions are in NCBI Build 38 coordinates.

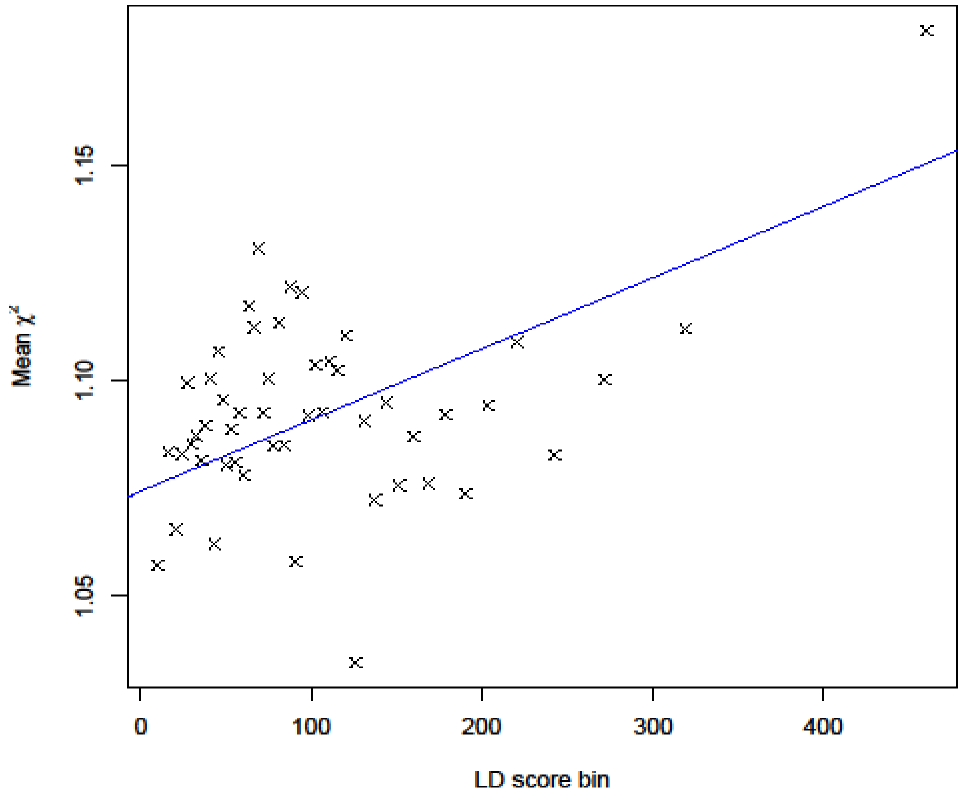

Supplementary Figure 3. **Relationship between LD score regression and genomic control inflation factor for the endometriosis dataset**

LD score regression plot for endometriosis with each point representing a LD score quantile. The x-axis coordinates represent the mean LD score of a quantile and the y-axis the mean $\chi 2$ statistic of the quantile. The blue line is the LD score regression line.

Supplementary Table 1. Association results for variant previously reported to associate with endometriosis in GWAS studies

SNP	Chr	Pos hg38	Risk allele	Case selection	Meta-analysis results[a] *P*-meta	Meta-analysis results[a] OR (95% CI)	Icelandic data[b] RAF[c]	Icelandic data[b] *P*-value	Icelandic data[b] OR (95% CI)	Nearest gene
rs7521902	1	22164231	A	All	1.8×10^{-15}	1.18 (1.13-1.23)	28.0	0.034	1.09 (1.01-1.18)	*WNT4*
				III/IV only	1.8×10^{-10}	1.25 (1.16-1.33)		0.025	1.16 (1.02-1.32)	
				III/IV vs I/II				0.10	1.18 (0.97-1.44)	
rs13394619	2	11587381	G	All	4.5×10^{-8}	1.13 (1.07-1.20)	51.4	7.4×10^{-7}	1.20 (1.12-1.29)	*GREB1*
				III/IV only	2.1×10^{-3}	1.18 (1.11-1.24)		0.032	1.14 (1.01-1.29)	
				III/IV vs I/II				0.75	0.97 (0.80-1.17)	
rs4141819	2	67637543	C	All	2.1×10^{-4}	1.08 (1.04-1.12)	29.0	0.050	1.08 (1.00-1.17)	*ETAA1*
				III/IV only	6.9×10^{-6}	1.16 (1.09-1.24)		0.0072	1.19 (1.05-1.35)	
				III/IV vs I/II				0.07	1.19 (0.99-1.44)	
rs1250248	2	215422370	A	All	1.1×10^{-4}	1.11 (1.04-1.18)	33.9	0.0015	1.13 (1.05-1.22)	*FN1*
				III/IV only	8.0×10^{-8}	1.26 (1.16-1.38)		0.055	1.13 (1.00-1.28)	
				III/IV vs I/II				0.90	1.01 (0.86-1.18)	
rs6734792	2	150768368	C	All	9.7×10^{-5}	1.10 (1.06-1.16)	35.5	0.40	1.03 (0.96-1.10)	*RND3*
				III/IV only	6.5×10^{-5}	1.10 (1.05-1.15)		0.36	1.06 (0.94-1.20)	
				III/IV vs I/II				0.17	1.13 (0.95-1.35)	
rs7739264	6	19785357	T	All	1.9×10^{-10}	1.11 (1.08-1.15)	49.4	0.12	1.06 (0.98-1.14)	*ID4*
				III/IV only	1.2×10^{-8}	1.20 (1.13-1.28)		0.10	1.10 (0.98-1.23)	
				III/IV vs I/II				0.20	1.12 (0.94-1.33)	
rs12700667	7	25862019	A	All	1.9×10^{-9}	1.13 (1.08-1.17)	72.7	0.032	1.10 (1.01-1.20)	*NFE2L3*
				III/IV only	4.5×10^{-8}	1.22 (1.14-1.31)		0.0071	1.20 (1.05-1.37)	
				III/IV vs I/II				0.034	1.23 (1.02-1.49)	
rs7798431	7	25821192	G	All	5.4×10^{-9}	1.13 (1.09-1.18)	76.5	0.0094	1.12 (1.03-1.22)	*NFE2L3*
				III/IV only	9.7×10^{-8}	1.24 (1.14-1.33)		0.0046	1.23 (1.07-1.42)	
				III/IV vs I/II				0.084	1.20 (0.98-1.48)	
rs1537377	9	22169701	C	All	1.0×10^{-8}	1.12 (1.08-1.17)	42.2	0.034	1.08 (1.01-1.16)	*CDKN2B-AS1*

				III/IV only	8.1×10^{-8}	1.18 (1.11-1.26)		0.0017	1.21 (1.07-1.36)	
				III/IV vs I/II				0.057	1.19 (0.99-1.42	
rs1333049	9	22125504	G	All	0.25	1.04 (0.98-1.10)	55.3	0.00084	1.13 (1.05-1.21)	*CDKN2B-AS1*
				III/IV only	0.55	1.03 (0.94-1.12)		0.0085	1.18 (1.04-1.33)	
				III/IV vs I/II				0.88	1.01 (0.89-1.15)	
rs10859871	12	95318100	C	All	4.8×10^{-15}	1.18 (1.13-1.22)	29.9	0.047	1.08 1.00-1.17)	*VEZT*
				III/IV only	6.8×10^{-7}	1.19 (1.11-1.27)		0.0048	1.20 (1.06-1.36)	
				III/IV vs I/II				0.51	1.06 (0.89-1.26)	

[a] data based on 11,506 endometriosis cases and 32,678 controls of European and Japanese origin for variants that have previously been reported with genome wide significance in endometriosis GWAS studies (from Rahmioglu, N. et al. *Hum. Reprod. Update* **20,** 702–716 (2014)). [b] association results obtained in the Icelandic population sample from the current analysis for all endometriosis cases (N = 1,840 cases and 129,016 controls), endometriosis cases with stage III/IV disease (N = 688 cases and 123,526 controls) and endometriosis cases with stage III/IV disease tested against cases with stage I/II disease (N = 688 cases and 620 controls). [c] RAF = risk allele frequency in the Icelandic data.

Supplementary Table 2. Follow up of variants with *P* < $1x10^{-6}$ in GWAS of all endometriosis cases.

					Discovery[a]				Follow up[b]			
Marker	Chr	Position (hg38)	A_{min}	A_{maj}	MAF	*P*-value	OR[c]	Proxy tested (r^2)	*P*-value	OR	Nearest gene	Coding effect
rs118137751	7	113897738	A	G	4.18	9.8E-09	1.59		0.23	0.77	*PPP1R3A*	intron
rs519664	9	15246654	T	C	20.42	1.6E-08	1.28		0.0090	1.30	*TTC39B*	intron
rs4497965	22	39448781	T	C	12.70	1.1E-07	1.32		0.95	1.01	*MGAT3*	intergenic
rs17773813	4	55142802	A	G	31.76	1.3E-07	0.81		1.3E-05	0.67	*KDR*	intergenic
rs7826510	8	5935362	G	T	15.44	3.2E-07	1.28	rs7822296 (1)	0.98	0.99	*LOC100287015*	intergenic
rs767233639	17	1934814	G	GC	0.05	6.0E-07	9.19				*RTN4RL1*	3’ UTR
rs1471289	5	93349720	C	G	25.26	9.3E-07	0.80		0.59	1.05	*NR2F1-AS1*	intergenic

[a] association results for variants at all novel loci with *P* < $1x10^{-6}$ in the Icelandic analysis for all endometriosis (N = 1,840 cases and 129,016 controls).

[b] association results in the Danish follow up samples (N = 514 cases and 749 controls).

[c] OR: Odds ratio reported on the minor allele; [d] NP: Not polymorphic

Supplementary Table 3. Association with disease severity for variants in linkage disequilibrium with rs767233639

Name	Pos chr17	A_{min}	A_{maj}	MAF %	Endometriosis Effect A_{min}	Endometriosis *P*-value	Stage III/IV Effect A_{min}	Stage III/IV *P*-value	Stage I/II Effect A_{min}	Stage I/II *P*-value	Stage III/IV vs I/II Effect A_{min}	Stage III/IV vs I/II *P*-value
chr17:1155537:0	1155537	A	G	0.021	15.75	1.1E-06	29.04	1.0E-06	4.84	0.21	2.63	0.23
rs779385919	1270250	T	C	0.021	15.6	1.2E-06	28.79	1.1E-06	4.76	0.22	2.63	0.23
chr17:1437418:0	1437418	GA	G	0.021	15.67	1.1E-06	28.84	1.1E-06	4.82	0.22	2.63	0.23
rs186322894	1440971	A	C	0.021	15.67	1.1E-06	28.84	1.1E-06	4.82	0.22	2.63	0.23
rs191466924	1479805	A	G	0.025	11.52	7.3E-06	22.02	3.8E-06	3.80	0.28	2.63	0.23
rs767233639	1934814	G	GC	0.05	9.19	6.0E-07	15.45	7.9E-07	5.31	0.064	2.12	0.27
rs61169988	3182558	A	AAAG	0.019	13.15	6.1E-05	21.46	0.00021	5.63	0.18	1.81	0.52
rs778764728	3662028	T	C	0.02	15.33	6.1E-06	25.94	1.4E-05	5.33	0.19	2.18	0.36
rs771535120	5921572	G	A	0.032	9.51	8.0E-05	17.60	6.8E-05	3.58	0.30	2.18	0.36

Variants correlated with rs767233639 ($R^2 > 0.2$) in a region spanning the p-terminus of chr17 up to 10 Mb upstream of rs767233639 that associated with endometriosis with P< 0.01 were tested for association with endometriosis severity.

Supplementary Table 4. Conditional analysis of variants in linkage disequilibrium with rs767233639

										Adjusting for rs767233639		Adjusting rs767233639 for covariates	
Name	Pos chr17	A_{min}	A_{maj}	MAF %	Info	Nearest gene	Coding effect	R^2	Effect Amin	Unadjusted *P*-value	Adjusted *P*-value	Unadjusted *P*-value	Adjusted *P*-value
chr17:1155537:0	1155537	A	G	0.021	0.99	*ABR*	intron	0.42	15.75	1.1E-06	0.18	6.0E-07	0.037
rs779385919	1270250	T	C	0.021	0.99	*BHLHA9*	upstream	0.42	15.60	1.2E-06	0.19	6.0E-07	0.036
chr17:1437418:0	1437418	GA	G	0.021	0.99	*CRK*	intron	0.42	15.67	1.1E-06	0.19	6.0E-07	0.036
rs186322894	1440971	A	C	0.021	0.99	*CRK*	intron	0.42	15.67	1.1E-06	0.19	6.0E-07	0.036
rs191466924	1479805	A	G	0.025	0.99	*MYO1C*	intron	0.30	11.52	7.3E-06	0.26	6.0E-07	0.014
rs767233639	1934814	G	GC	0.05	0.96	*RTN4RL1*	3'UTR	1.0	9.19	6.0E-07	1	6.0E-07	1
rs61169988	3182558	A	AAAG	0.019	1.00		intergenic	0.42	13.15	6.1E-05	0.66	6.0E-07	0.0017
rs778764728	3662028	T	C	0.02	1.00	*CTNS*	3'UTR	0.35	15.33	6.1E-06	0.27	6.0E-07	0.0084
rs771535120	5921572	G	A	0.032	0.98	*LOC339166*	intron	0.21	9.51	8.0E-05	0.33	6.0E-07	0.0014

Variants correlated with rs767233639 ($R^2 > 0.2$) in a region spanning the p-terminus of chr17 up to 10 Mb upstream of rs767233639 that associated with endometriosis with P< 0.01 were tested for association before and after adjusting for the effect of rs767233639. Association of rs767233639 was also tested by adjusting for each of the 8 correlated variants.
